# Loss of *DEPTOR* in renal tubules protects against cisplatin-induced acute kidney injury

**DOI:** 10.1038/s41419-018-0483-3

**Published:** 2018-04-18

**Authors:** Caixia Wang, Huaiqian Dai, Zhi Xiong, Qiancheng Song, Zhipeng Zou, Mangmang Li, Jing Nie, Xiaochun Bai, Zhenguo Chen

**Affiliations:** 1 The State Key Laboratory of Organ Failure Research, Department of Cell Biology, School of Basic Medical Sciences, Southern Medical University, Guangzhou, 510515 China; 2 The State Key Laboratory of Organ Failure Research, Department of Nephrology, Nanfang Hospital, Southern Medical University, Guangzhou, 510515 China

## Abstract

DEP domain containing mTOR-interacting protein (DEPTOR) was originally identified as an in vivo dual inhibitor of mechanistic target of rapamycin (mTOR). It was recently reported to be involved in renal physiology and pathology in vitro; however, its detailed roles and mechanisms in vivo are completely unknown. We observed that DEPTOR expression in the kidney was markedly increased on day 3 after cisplatin treatment, at which time cell apoptosis peaked, implicating DEPTOR in cisplatin-induced acute kidney injury (AKI). We then used the Cre–LoxP system to generate mutant mice in which the *DEPTOR* gene was specifically deleted in the proximal tubule cells. *DEPTOR* deficiency did not alter the renal histology or functions in the saline-treated group, indicating that DEPTOR is not essential for kidney function under physiological conditions. Interestingly, *DEPTOR* deletion extensively preserved the renal histology and maintained the kidney functions after cisplatin treatment, suggesting that the absence of DEPTOR ameliorates cisplatin-induced AKI. Mechanistically, DEPTOR modulated p38 MAPK signaling and TNFα production in vivo and in vitro, rather than mTOR signaling, thus moderating the inflammatory response and cell apoptosis induced by cisplatin. Collectively, our findings demonstrate the roles and mechanisms of DEPTOR in the regulation of the renal physiology and pathology, and demonstrate that the loss of *DEPTOR* in the proximal tubules protects against cisplatin-induced AKI.

## Introduction

Acute kidney injury (AKI), which often results from ischemia/reperfusion, sepsis, or nephrotoxins, is a major kidney disease characterized by the rapid loss of renal function, leading to the accumulation of metabolic wastes and imbalances in electrolytes and body fluids^1–3^. It is associated with high rates of morbidity and mortality. AKI is also frequently associated with and may contribute to the development of chronic kidney disease (CKD)^4,5^.

Cisplatin is a widely used chemotherapeutic drug, with major adverse effects in the kidney, inducing AKI^6,7^. The nephrotoxicity of cisplatin mainly involves the death of the tubule cells, including both apoptosis and necrosis, because it activates complex signaling pathways^8,9^. A robust inflammatory response is also stimulated, further exacerbating the renal tissue damage^10^. Cisplatin may also injure the renal vasculature, reducing blood flow and inducing ischemic injury in the kidney^10,11^. It is well recognized that the renal tubules are the major sites of cell injury and death during cisplatin nephrotoxicity. Recent studies have indicated that cell apoptosis occurs in both the distal and proximal tubules, although the majority occurs in the proximal tubules^12^. Cisplatin nephrotoxicity is a very complex multifactorial process that includes oxidation, inflammation, genotoxic damage, and cell-cycle arrest, and a greater understanding of its mechanisms may lead to the development of novel renoprotective interventions^10^.

DEP domain containing mTOR-interacting protein (DEPTOR, also known as DEPDC6) binds to both mechanistic target of rapamycin complex 1 (mTORC1) and mTORC2, inhibiting their activities^13^. DEPTOR expression is low in most cancers, consistent with the activation of the mTORC1 and mTORC2 pathways in many human cancers. However, DEPTOR is surprisingly strongly expressed in some cancers, including multiple myeloma. The high DEPTOR expression in these cells reduces their mTORC1 activity and activates protein kinase B (Akt/PKB) signaling via a negative feedback loop from S6 kinase 1 (S6K1) to phosphoinositide-3 kinase (PI3K), promoting cell survival^13^. DEPTOR can be ubiquitinated and degraded by SCF^β-TrCP^ E3 ligase after its phosphorylation. Recent studies have shown that many kinases, including mTOR, S6K, 90-kDa ribosomal S6 kinase 1 (RSK1), casein kinase I (CK1), and p38γ/δ, can phosphorylate DEPTOR, promoting its ubiquitination and degradation^14–17^.

Several in vitro studies have reported the roles of DEPTOR in the renal physiology and pathology. In cultured proximal tubule epithelial cells, the downregulation of DEPTOR by transforming growth factor β (TGFβ) recruited mTORC1, but not mTORC2, thus enhancing the expression of the *Col1α2* gene and promoting the development of TGFβ-induced renal fibrosis^18^. In renal glomerular mesangial cells, DEPTOR suppression by Smad3 in response to TGFβ aggravated mesangial cell hypertrophy^19^. However, the roles and mechanisms of DEPTOR in the regulation of the physiological and pathological renal processes in vivo are unknown.

Therefore, in this study, we developed a mouse model in which the *DEPTOR* gene was specifically deleted in the proximal tubules to investigate the roles and regulatory mechanisms of DEPTOR in proximal tubule cell functions and cisplatin-induced AKI in vivo. We demonstrate that *DEPTOR* knockout (cKO) protected the proximal tubules from cisplatin-induced AKI, and that this protection was probably mediated by reduced cell death through the inhibition of p38 MAPK signaling and the production of tumor necrosis factor α (TNFα).

## Results

### DEPTOR expression in proximal tubules is increased in mice with cisplatin-induced AKI

**Fig. 1:**
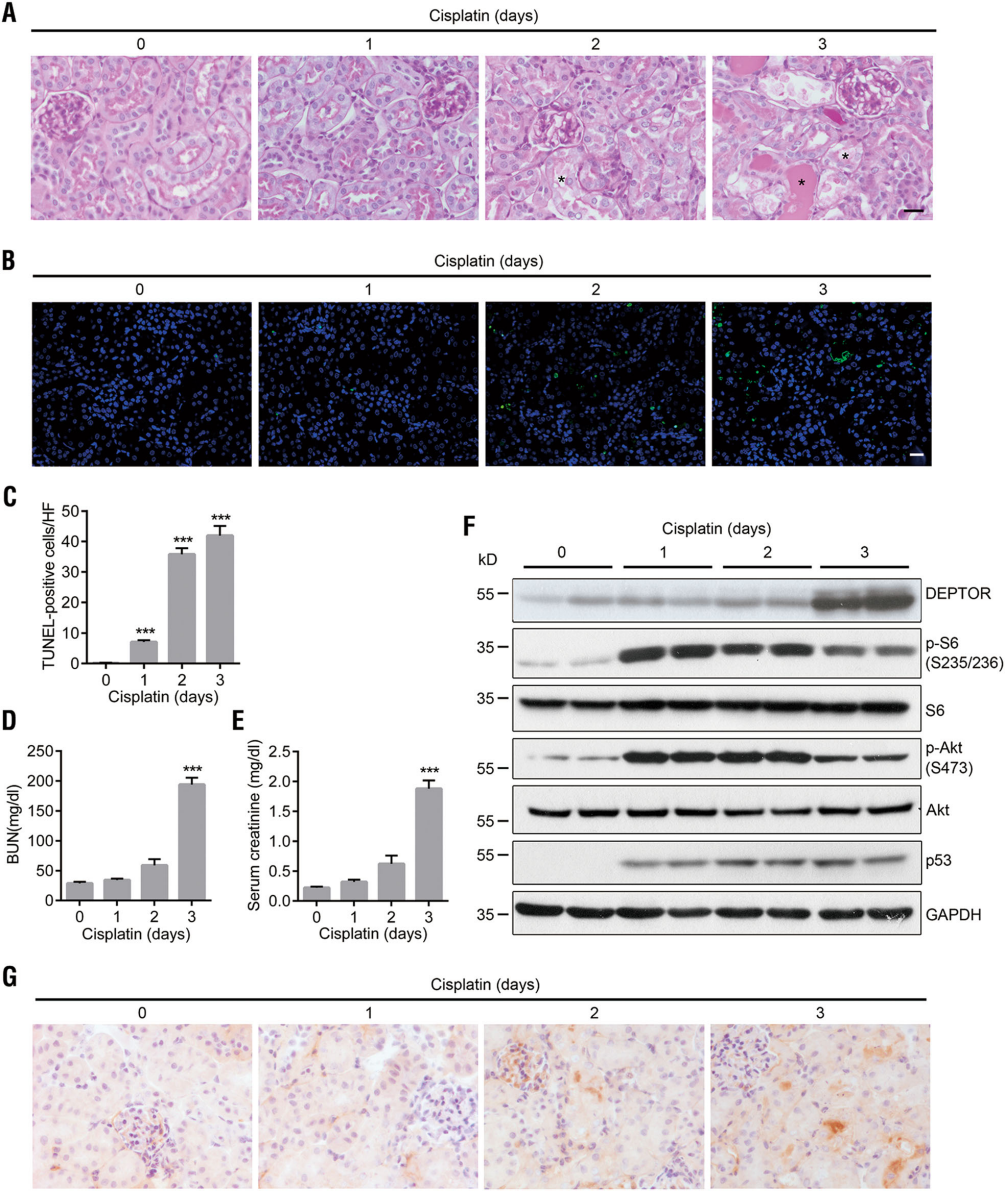
Induction of DEPTOR expression in proximal tubules by cisplatin. **a** Kidney histology in 8-week-old C57BL/6 mice on days 0–3 after intraperitoneal injection with cisplatin (a single dose of 20 mg/kg), detected with PAS staining. The asterisks indicate tubules with necrosis and cast formation. **b** Renal cell apoptosis was assessed with a fluorometric TUNEL assay (green). Nuclei were stained with DAPI (blue). **c** Quantitative data for apoptotic cells. Data are expressed as the number of TUNEL-positive cells per high-magnification field (HF, ×400). **d** Assessment of BUN and (**e**) serum creatinine levels. **f** Western blotting of p-S6 (S235/S236), p-Akt (S473), and p53. Protein samples were extracted from the renal cortex. **g** IHC staining of DEPTOR. Scale bar = 20 μm. Bars indicate mean values ± SEM. *n* = 6. ***P < 0.001 compared with day 0

To determine the role of DEPTOR in cisplatin-induced AKI in vivo, we injected C57 mice intraperitoneally with cisplatin and detected DEPTOR expression in their kidney tissues. On day 2 after cisplatin treatment, periodic acid–schiff (PAS) staining showed that the renal cortices had begun to display slight histological changes, including brush border loss, tubule dilation, and the collapse of some tubules (Fig. 1a). On day 3, the renal cortices showed widespread necrosis, cast formation, and brush border disruption (Fig. 1a). A terminal deoxynucleotidyl-transferase-mediated deoxyuridine triphosphate nick end labeling (TUNEL) analysis also showed a time-dependent increase in apoptotic cells during the cisplatin treatment (Figs. 1b,c). Marked increases in blood urea nitrogen (BUN) and serum creatinine on day 3 clearly indicated cisplatin nephrotoxicity (Figs. 1d,e) and the successful generation of a model of AKI.

A western blotting analysis showed that DEPTOR expression in the renal cortex was almost constant from day 0 (saline group) to day 2 after cisplatin treatment, but increased sharply on day 3, indicating that DEPTOR is involved in cisplatin-induced AKI, particularly in its later stage (Fig. 1f). Therefore, we used AKI mice on day 3 after cisplatin treatment for the subsequent experiments. The induction of phospho-S6 (p-S6, S235/S236) and p-Akt (S473) peaked on days 1–2, and then declined on day 3 (Fig. 1f), consistent with the previously observed inhibition of mTORC1/2 by DEPTOR. It is noteworthy that DEPTOR expression was synchronous with cell apoptosis (Figs. 1b,c). Immunohistochemical (IHC) staining also showed that DEPTOR expression was weak in the normal proximal tubules, but was clearly enhanced after cisplatin treatment (Fig. 1g). These data suggest that DEPTOR is a key mediator of cisplatin-induced AKI.

### Generation of mutant mice with proximal tubule-specific deletion of *DEPTOR*

Because the proximal tubules are the tubule segment most vulnerable to cisplatin toxicity, to clarify the roles of DEPTOR in the pathology of AKI in vivo we used the Cre–LoxP system to generate conditional knockout (cKO) mice in which the *DEPTOR* gene was specifically deleted in the proximal tubule cells (Supplemental Fig. 1). No significant difference was observed in the bodyweight, kidney weight, or renal functions and morphology between the adult cKO mice and their littermate controls (Supplemental Fig. 2), suggesting that DEPTOR is not essential for kidney development and function under physiological conditions.

### Loss of *DEPTOR* in proximal tubules ameliorates cisplatin-induced AKI

**Fig. 2:**
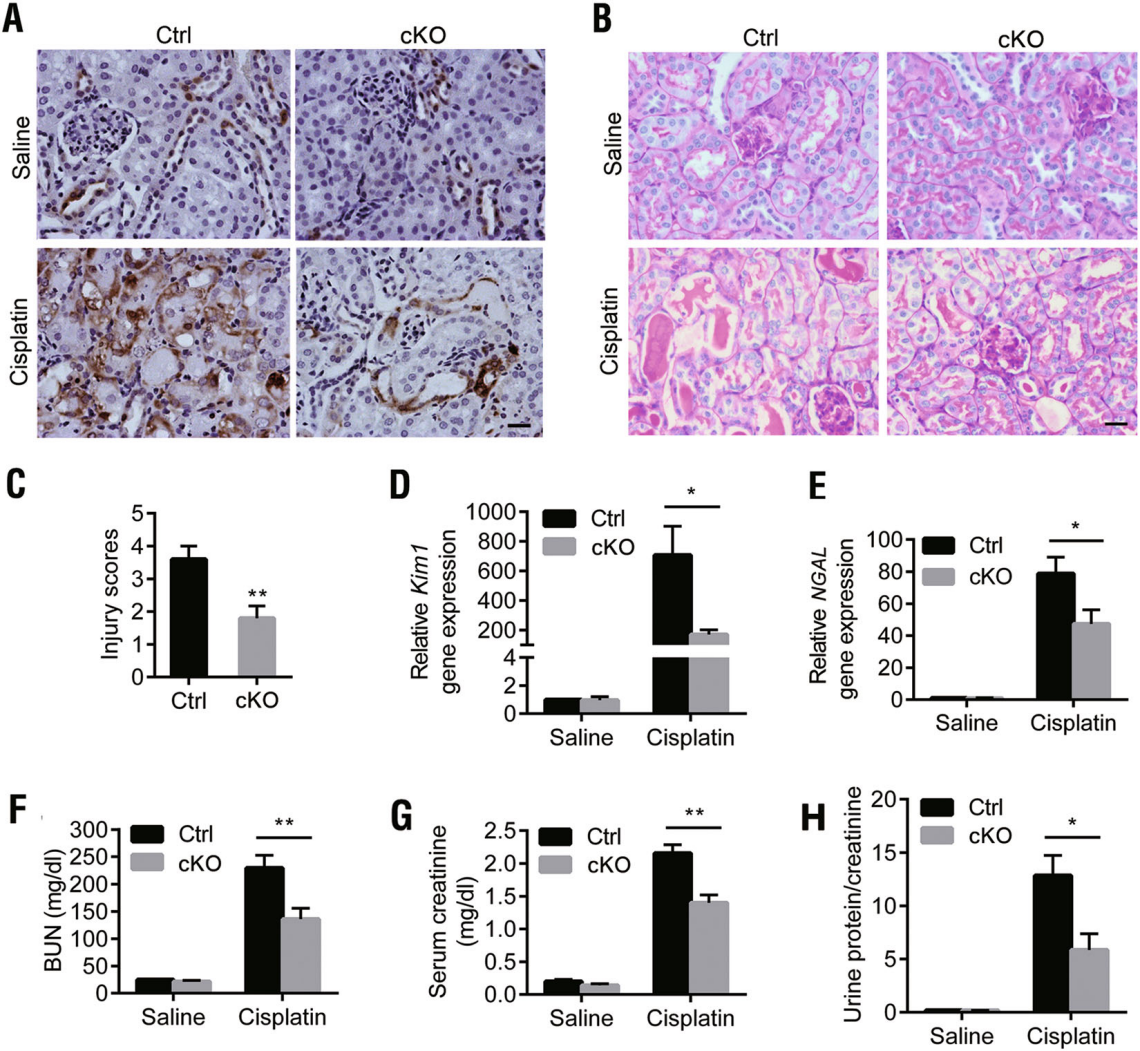
*DEPTOR* deletion in proximal tubules ameliorated cisplatin-induced AKI. **a** IHC staining of DEPTOR in control and *DEPTOR* cKO mice after treatment with saline or cisplatin. **b** PAS staining of kidney tissues from control and *DEPTOR* cKO mice after treatment with saline or cisplatin. **c** Injury scores for PAS-stained kidney sections. *n* = 6. **d** qPCR analysis of *Kim1* mRNA levels. **e** qPCR analysis of *NGAL* mRNA levels. **f** BUN and (**g**) serum creatinine levels, and (**h**) urinary protein/creatinine ratio in the paired mice. BUN, creatinine and urinary protein were measured with the IDEXX Catalyst Chemistry Analyzer. *n* = 6–11. All samples were from the mice on day 3 after cisplatin treatment. *n* = 6. Bars indicate mean values ± SEM. *P < 0.05; **P < 0.01. Scale bar = 20 μm. *KIM1* kidney injury molecule 1, *NGAL* neutrophil gelatinase-associated lipocalin; both are markers for acute renal tubule injury

We next investigated whether the deletion of *DEPTOR* in the proximal tubules affects cisplatin-induced AKI. IHC staining showed that the induction of DEPTOR after cisplatin treatment in the control group (Cis-Ctrl) was largely compromised by *DEPTOR* deletion (Cis-cKO; Fig. 2a). PAS staining showed a substantial loss of the brush border, more cast formation, tubule cell death, and detachment in the Cis-Ctrl mice, which were greatly counteracted in the Cis-cKO mice (Figs. 2b,c). Kidney injury also enhanced the transcription of kidney injury molecule 1 (*Kim1*) and neutrophil gelatinase-associated lipocalin (*NGAL*), the markers for acute renal tubule injury, both of which were reversed by *DEPTOR* deficiency (Figs. 2d, e), suggesting that kidney injury was attenuated in the Cis-cKO mice. The administration of cisplatin caused sharp increases in blood urine nitrogen (BUN), serum creatinine, and the urinary protein/creatinine ratio in the Cis-Ctrl mice, indicating the severe impairment of renal functions, which were very largely maintained by the deletion of *DEPTOR* in the Cis-cKO mice (Figs. 2f–h). These results together demonstrate that the loss of *DEPTOR* in the proximal tubules protects the kidney against cisplatin-induced AKI.

### Loss of *DEPTOR* inhibits proximal tubule cell apoptosis in cisplatin-induced AKI

**Fig. 3:**
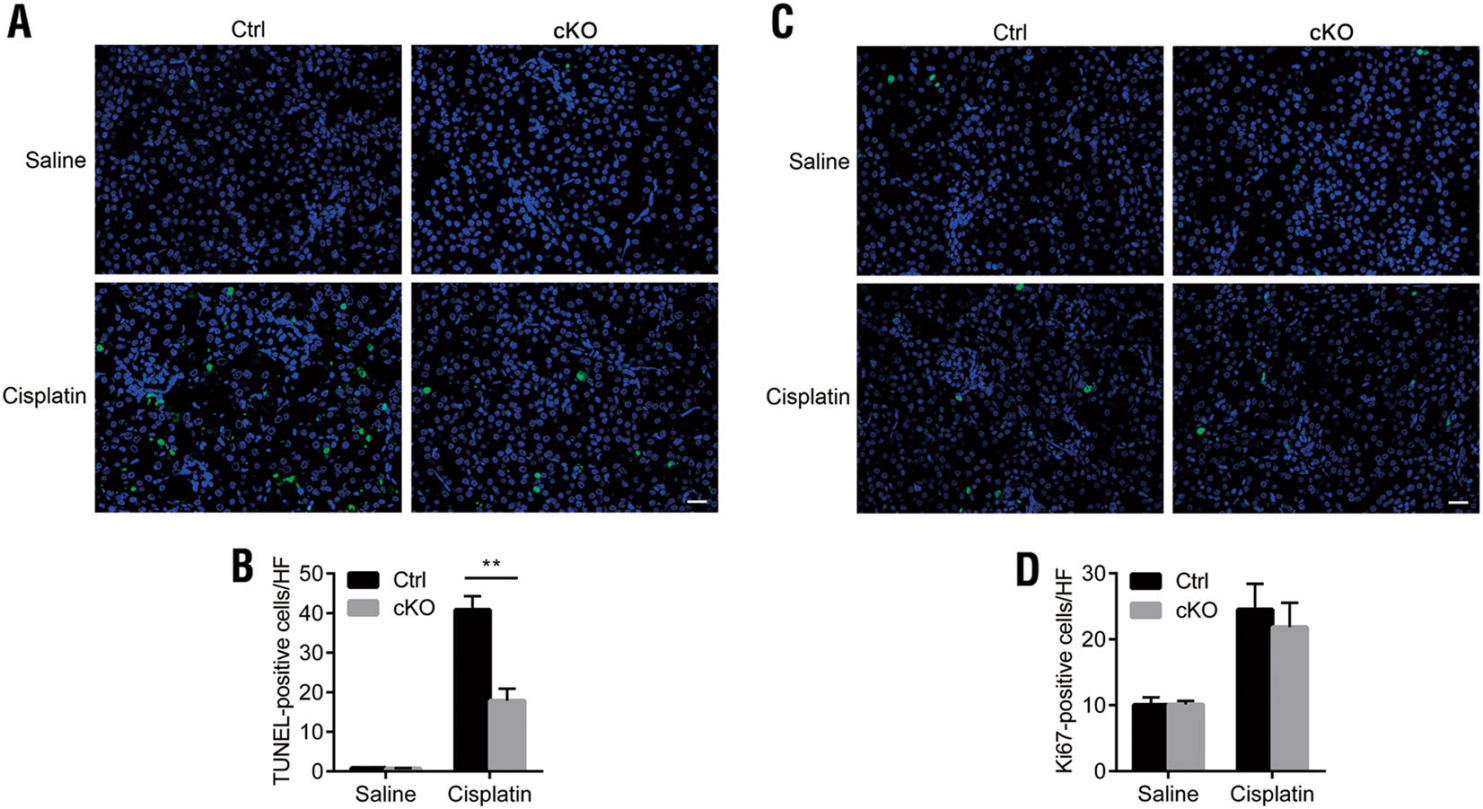
Cell apoptosis and cell proliferation in the kidneys of control and *DEPTOR* cKO mice before and after cisplatin-induced AKI. **a** Cell apoptosis analysis with a fluorometric TUNEL assay (green). Nuclei were stained with DAPI (blue). **b** Quantitative data for TUNEL-positive cells. Data are expressed as the number of TUNEL-positive signals per field (×400). **c** Cell proliferation analysis with Ki67 immunofluorescence (green). **d** Quantitative data for Ki67-positive cells. Data are expressed as the number of Ki67-positive signals per field (×400). All samples were from the mice on day 3 after cisplatin treatment. Scale bar = 20 μm. Bars indicate mean values ± SEM. *n* = 6. **P < 0.01

Previous studies have shown that cell apoptosis is an important form of tubule cell death, and the proliferative response facilitates renal repair and regeneration after injury^20^. To determine the mechanisms underlying the protective effect of *DEPTOR* cKO against cisplatin-induced AKI, we examined cell apoptosis and proliferation in the kidney after cisplatin treatment, using a TUNEL assay and Ki67-immunofluorescent staining, respectively. Negligible TUNEL-positive signals were detected in the saline-treated kidneys from both control and cKO mice, suggesting that the deletion of *DEPTOR* from proximal tubule cells does not affect cellular apoptosis under physiological conditions (Figs. 3a, b). However, after cisplatin treatment, the number of TUNEL-positive cells was clearly increased in the Cis-Ctrl kidneys, whereas it was ~60% lower in the Cis-cKO kidneys, suggesting that silencing *DEPTOR* increases the resistance of proximal tubule cells to cell death induced by cisplatin (Figs. 3a, b).

Ki67-immunofluorescent staining showed few Ki67-positive cells in the renal tubules from the saline-treated control or cKO mice (Figs. 3c, d). However, after treatment with cisplatin, the number of Ki67-positive cells was strikingly increased in both the Cis-Ctrl and Cis-cKO mice, and these increases did not differ significantly (Figs. 3c, d). This result suggests that *DEPTOR* deficiency in the proximal tubules does not affect the proliferation of tubule cells under physiological conditions or during cisplatin treatment.

### *DEPTOR* deletion attenuates cisplatin-induced AKI but not predominantly via the mTOR signaling pathway

**Fig. 4:**
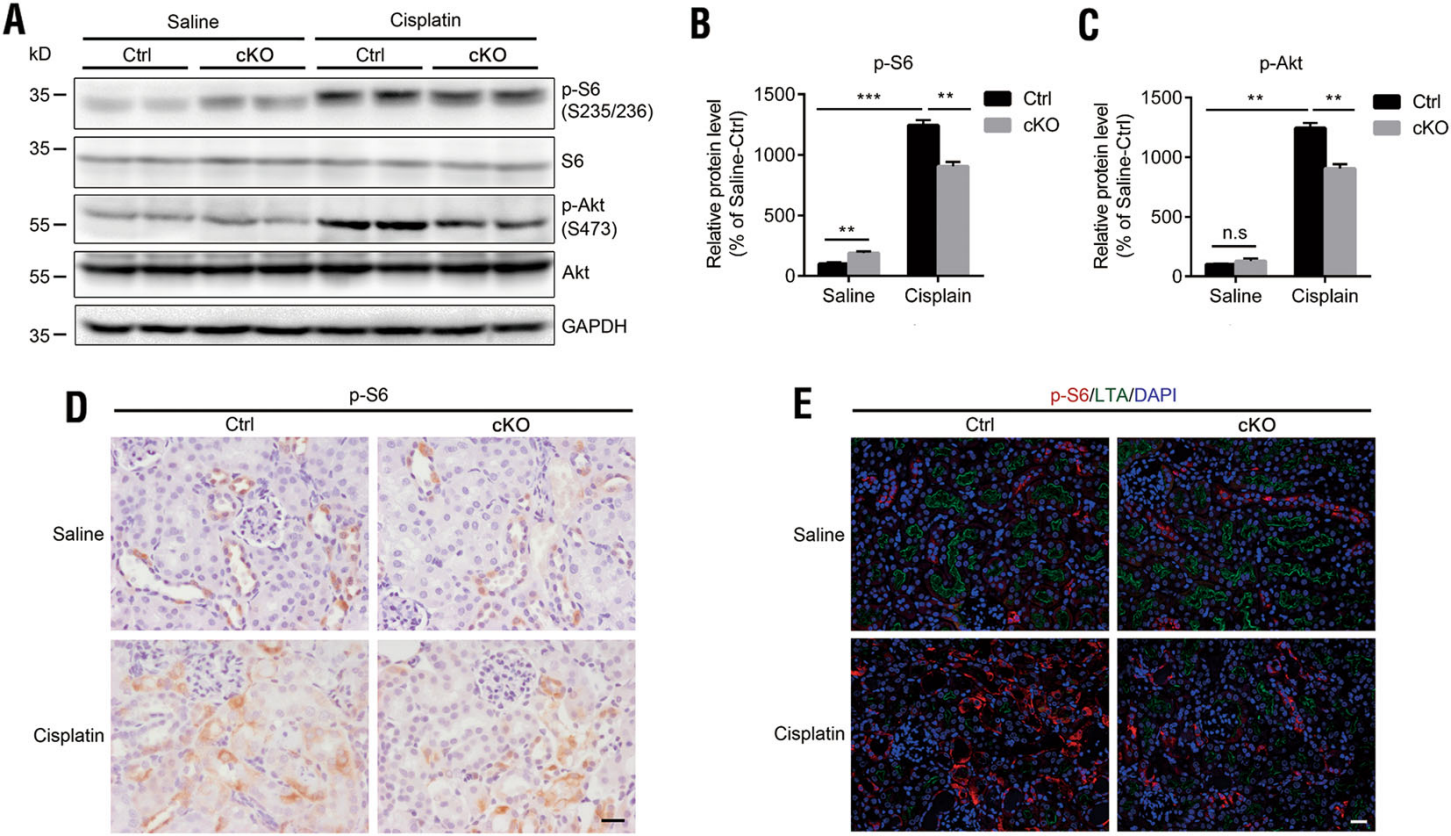
Expression of p-S6 (S235/S236) and p-Akt (S473) in control and DEPTOR cKO kidneys after cisplatin-induced AKI. **a** Western blotting of p-S6 (S235/S236) and p-Akt (S473) in the kidneys of cKO and control mice after cisplatin treatment. **b** Quantification of p-S6 and (**c**) p-Akt in the results of (**a**). Protein levels were normalized against GAPDH. Bars indicate mean values ± SEM. *n* = 5. **P < 0.01; ***P < 0.001; n.s., no significant difference. **d** IHC staining of p-S6 in cKO and control kidneys after cisplatin treatment. Scale bar = 20 μm. **e** Co-immunofluorescent staining for p-S6 and LTA in the kidneys of cKO and control mice after cisplatin treatment. Nuclei were stained with DAPI (blue). LTA, *Lotus tetragonolobus* lectin, is a marker of proximal tubules. All samples were from the mice on day 3 after cisplatin treatment. Scale bar = 50 μm

Because DEPTOR binds to both mTORC1 and mTORC2 and inhibits their activities, we determined the levels of p-S6 (S235/236) and p-Akt (S473), common downstream targets of mTORC1 and mTORC2 signaling, respectively. A western blotting analysis showed that the expression of p-S6 but not p-Akt was slightly increased in the cKO kidneys after saline treatment; however, both of them were lower in the Cis-cKO mice than in the Cis-Ctrl mice (Figs. 4a–c). This phenomenon induced an assumption that DEPTOR functions independently of the mTOR signaling pathway in cisplatin-induced AKI. To explore this possibility, we used IHC staining to detect p-S6, and found it was barely detectable in the proximal tubules in the both saline-treated groups (Fig. 4d). This was further confirmed by co-immunofluorescent staining for p-S6 and *Lotus tetragonolobus* lectin (LTA), a marker of proximal tubule cells (Fig. 4e). However, the increase in p-S6 in the Cis-Ctrl kidneys did not localize to the proximal tubules, in which *DEPTOR* was deleted; therefore, its lower levels in the Cis-cKO kidneys may be attributable to the excessive apoptosis of the cells expressing intact DEPTOR (Fig. 4e). Together, these results suggest that the protective effects of *DEPTOR* deletion in the proximal tubule cells in vivo are not predominantly mediated by the mTOR signaling pathway.

### p38 MAPK is involved in the regulation of cisplatin-induced AKI by DEPTOR in vivo

**Fig. 5:**
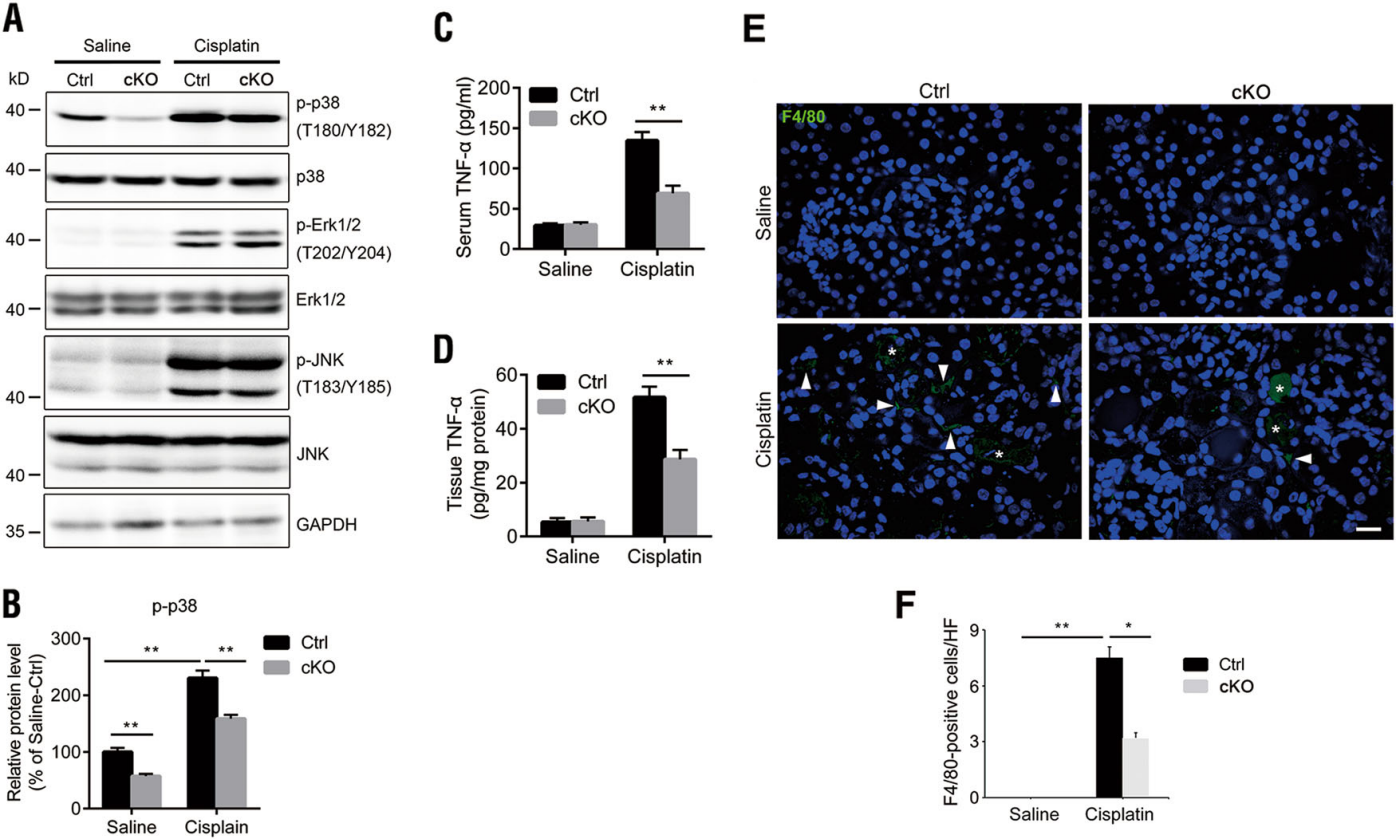
*DEPTOR* deletion in renal proximal tubules inhibited p38 activity and TNFα production. **a** Western blotting analysis of p-ERK (T202/Y204), p-JNK (T183/Y185), and p-p38 (T180/Y182) in the renal cortices of control and *DEPTOR* cKO mice after treatment with saline or cisplatin. **b** Quantification of p-p38 in the results of (**a**). Protein levels were normalized against GAPDH. **c** ELISA of serum TNFα levels in indicated groups. **d** ELISA of TNFα levels in renal cortices in indicated groups. **e** Representative micrographs showing immunofluorescent staining for F4/80 among groups as indicated. White triangle indicates positive staining (compact and specific); white asterisk indicates false-positive staining (diffused and background signals in tubule lumen). **f** Quantification of F4/80-positive cells. All samples were from the mice on day 3 after cisplatin treatment. Scale bar = 20 μm. Bars indicate mean values ± SEM. *n* = 5–7. *P < 0.05; **P < 0.01

The mitogen-activated protein kinase (MAPK) signaling pathway consists of several tiers of highly conserved serine/threonine protein kinases, and results in the terminal activation of p38 MAPK, extracellular signal-regulated kinase (ERK), and JUN N-terminal kinase (JNK) or stress-activated protein kinase (SAPK)^21^. p38, ERK, and JNK/SAPK are activated in various experimental models of cisplatin nephrotoxicity, and play critical roles in AKI^10^. Furthermore, DEPTOR is reported to regulate the activity of p38 or ERK in vitro^22,23^. Therefore, we investigated whether the MAPK pathway is involved in the regulation of DEPTOR in AKI. A western blotting analysis showed that p38, ERK, and JNK/SAPK were all activated after cisplatin treatment (Fig. 5a). Interestingly, the level of p-p38 (T180/Y182) was markedly reduced in the cKO kidneys under both physiological conditions and AKI, whereas the levels of p-ERK (T202/Y204) and p-JNK (T183/Y185) were similar in the control and cKO kidneys, under either physiological conditions or AKI (Figs. 5a, b). These results indicate that p38, but not ERK or JNK, is involved in the role of DEPTOR in cisplatin-induced AKI.

Previous studies have shown that p38 regulates TNFα expression in renal tubule cells and the consequent inflammatory response during cisplatin nephrotoxicity^24,25^. Therefore, we monitored the expression of TNFα in our model. The results showed comparable TNFα mRNA and protein levels in the saline-treated control and cKO kidneys. After cisplatin treatment, both of them were greatly enhanced in the kidneys, but were reduced in the Cis-cKO kidneys compared with Cis-Ctrl kidneys (Figs. 5c, d). Consistently, more F4/80-positive cells indicating macrophage infiltration were detected in the Cis-cKO group than in the Cis-Ctrl group (Figs. 5e, f). These results suggested that DEPTOR deficiency blocks p38 signaling and TNFα production, reducing the inflammatory response during AKI.

### DEPTOR regulates p38 MAPK activity in vitro

**Fig. 6:**
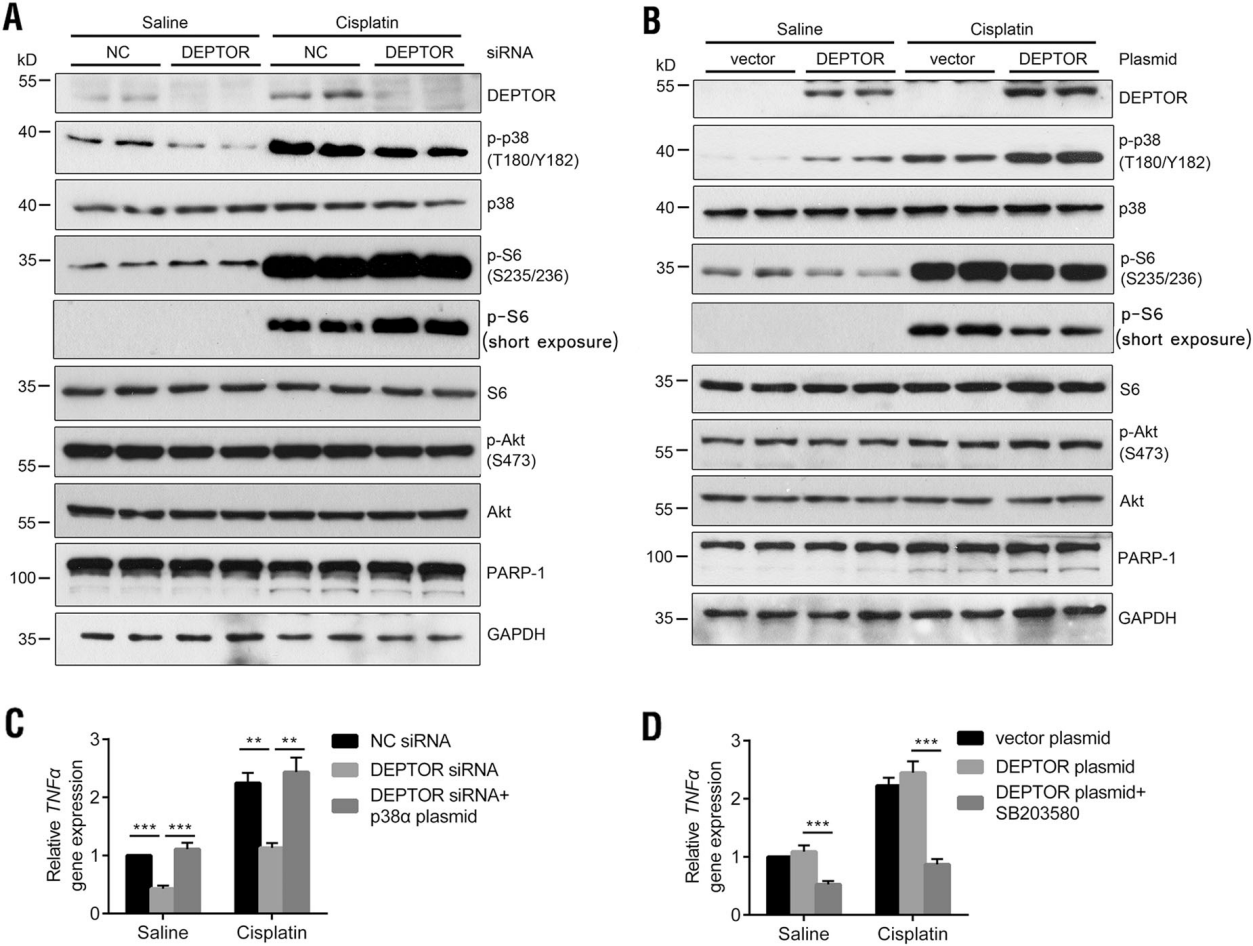
DEPTOR regulates p38 MAPK activity and TNFα secretion in cultured cells. **a** Immunoblotting analysis of p-p38, p-S6, p-Akt, and cleaved PARP-1 levels after *DEPTOR* knockdown by siRNA in HK-2 cells, with or without cisplatin treatment. In the cisplatin group, cells were incubated with 20 μM cisplatin for 24 h, the same below. **b** Immunoblotting analysis of p-p38, p-S6, p-Akt, and cleaved PARP-1 levels after *DEPTOR* overexpression in HK-2 cells. **c** qPCR analysis of *TNFα* mRNA levels after *DEPTOR* knockdown in HK-2 cells under saline or cisplatin condition, with or without p38 MAPKα overexpression. **d** qPCR analysis of *TNFα* mRNA levels after *DEPTOR* overexpression in HK-2 cells under saline or cisplatin condition, with or without a p38 MAPK inhibitor, SB203580 (50 µM). Bars indicate mean values ± SEM. *n* = 6. **P < 0.01

We next tested the modulation of p38 MAPK activity by DEPTOR in two cultured-cell models in vitro. In HK-2 cells, a human kidney proximal tubule epithelial cell line, DEPTOR expression was knocked down with small interfering RNA (siRNA) targeting the *DEPTOR* mRNA. Western blotting showed that the DEPTOR siRNA reduced the DEPTOR protein level by 70% relative to that in the negative control group, and significantly inhibited the activity of p38 in the absence or presence of cisplatin (Fig. 6a). Consistent with this, DEPTOR ectopically expressed in HK-2 cells promoted p38 phosphorylation in both the saline-treated and cisplatin-treated cells (Fig. 6b). Regulation of p38 by DEPTOR was also replicated in HEK293 cells (Supplemental Fig. 3). The expression of p-S6 was increased by *DEPTOR* knockdown and was decreased by *DEPTOR* overexpression in HK-2 cells, either under saline or cisplatin condition. While the level of p-Akt was hardly changed after altering the DEPTOR expression. This discrepancy between in vitro and in vivo data (Fig. 4a) may be due to a different response to cisplatin, or a negative feedback loop from S6K1 to PI3K/Akt^13^. In addition, western blotting analysis of cleaved PARP-1 demonstrated that DEPTOR promotes cisplatin-induced cell apoptosis in HK-2 cells (Figs. 6a,b). *DEPTOR* knockdown in HK-2 cells also reduced the *TNFα* mRNA level, but was reversed by ectopic expression of p38 (Fig. 6c). Consistently, increased *TNFα* mRNA level by *DEPTOR* overexpression was also compromised by a p38 inhibitor, SB203580 (Fig. 6d). Together, these results demonstrate that DEPTOR modulates p38 activity in response to cisplatin-induced AKI.

## Discussion

In this study, we first demonstrated that DEPTOR expression is significantly increased in the mouse kidney after treatment with cisplatin for 3 days, when tubule cell apoptosis peaked, suggesting that DEPTOR is involved in the process of cisplatin-induced AKI. By generating a mouse model with a proximal tubule-specific deletion of the *DEPTOR* gene, we showed that *DEPTOR* cKO antagonizes cisplatin-induced AKI, evident as improved renal histology and functions after *DEPTOR* deletion and cisplatin treatment. Both the in vivo and in vitro evidence demonstrates mechanistically that *DEPTOR* deficiency protects proximal tubule cells against the apoptosis induced by AKI by inhibiting p38 MAPK signaling and TNFα production.

DEPTOR contains two tandem DEP domains at its N terminus and a PDZ domain at its C terminus. It binds directly through its PDZ domain to the FAT domain of mTOR and blocks the kinase activities of both mTORC1 and mTORC2^13^. Recent studies of DEPTOR have focused on its roles in tumor development and progression, whereby it affects cell growth, apoptosis, autophagy, and the endoplasmic reticulum stress response^26,27^. However, the roles of DEPTOR in physiological processes in vivo have been unclear. Laplante and his colleagues first generated DEPTOR overexpression and knockout mice to investigate the roles of DEPTOR in energy metabolism in the white adipose tissue, mediobasal hypothalamus, pro-opiomelanocortin neurons, and liver^28–31^. In cultured proximal tubule epithelial cells, DEPTOR was shown to impede TGFβ-induced renal fibrosis by suppressing mTORC1 activity; however, its roles and mechanisms in tubule cells during AKI remained completely unknown^18^. We first determined the DEPTOR expression profile during cisplatin-induced AKI and observed that it peaked on day 3 after cisplatin treatment, suggesting that DEPTOR plays an important role in the pathology of cisplatin-induced AKI. We then used the Cre–LoxP system to create a mutant mouse in which the *DEPTOR* gene is specifically deleted in the proximal tubule cells. The *PEPCK*–Cre recombinase used in this study previously deleted the floxed gene in most (70–80%) proximal tubules, but not in other tubule segments^32^. We generate *DEPTOR*-flox mice and have demonstrated that the *DEPTOR* gene can be successfully deleted with the Cre recombinase. Our data show that the specific deletion of *DEPTOR* in the proximal tubules significantly improved the kidney functions, preserved the renal histology, and reduced cell apoptosis during cisplatin-induced AKI.

mTOR forms two distinct functional complexes. mTORC1 consists of mTOR, Raptor, DEPTOR, the TTI1/TEL2 complex, PRAS40, and mLST8, whereas mTORC2 comprises mTOR, Rictor, DEPTOR, the TTI1/TEL2 complex, mSIN1, mLST8, and Protor^33^. It has been reported that mTORC1-deficient animals created with the *Pax8rtTA*TetOCre Raptor^flox/flox^ model, in which all tubule segments are affected, showed markedly aggregated kidney damage after ischemia/reperfusion injury, attributed to increased cell apoptosis and a reduced proliferative response^20^. mTORC2-deficient mice created with the *Ksp*–Cre *Rictor*^flox/flox^ model, which has ~80% deletion efficiency in distal tubules, showed exacerbated kidney damage during cisplatin-induced AKI, mainly because cell survival was suppressed by the inactivation of Akt signaling and the inhibition of autophagy^34^. These two findings demonstrate that both mTORC1 and mTORC2 protect the kidney against AKI. Because DEPTOR is a natural dual inhibitor of both mTORs, its deletion should attenuate AKI. As expected, our results show that *DEPTOR* knockout in proximal tubule cells strongly protected the kidney functions and the renal histology after cisplatin treatment. However, the activities of both mTORC1 and mTORC2 in the cKO mice were negligibly increased, suggesting that DEPTOR regulates cisplatin-induced AKI in an mTOR-independent manner, which is confirmed in cultured proximal tubule epithelial cells.

We next demonstrated that p38 MAPK activity, but not ERK1/2 or JNK activity, declined markedly in the *DEPTOR* cKO mice after both saline and cisplatin treatments. However, in a study of vascular endothelial cells, DEPTOR inhibited the mTOR and ERK1/2 signaling pathways through independent mechanisms^22^. Consistent with the decline in p38 activity, a reduction in cell apoptosis was also observed in the cKO mice after cisplatin treatment. However, contrary to our finding, it was previously reported that in cervical squamous cell carcinoma cells, *DEPTOR* silencing activated p38 MAPK in an mTOR-independent manner, promoting cell apoptosis^23^. This discrepancy may be attributable to the heterogeneity of tumor cells and normal cells. Regarding the role of p38 in cisplatin nephrotoxicity, it has been suggested that instead of directly regulating tubule cell injury and death, p38 may promote TNFα production in renal tubule cells and the consequent inflammatory response during cisplatin nephrotoxicity^24,25^. TNFα is a key upstream regulator of the inflammatory response induced by cisplatin, which then mediates the expression of other proinflammatory chemokines and cytokines in AKI^35,36^. Consistent with this, we observed a significant decline in TNFα secretion and macrophage infiltration in the *DEPTOR* cKO mice after cisplatin treatment. Therefore, we inferred that DEPTOR silencing suppresses the action of TNFα by inhibiting p38, thus blocking the inflammatory response induced by cisplatin, which contributes to the attenuation of cisplatin nephrotoxicity.

In summary, we have shown that the deletion of *DEPTOR* in proximal tubule cells antagonizes cisplatin-induced AKI. The absence of DEPTOR in proximal tubule cells extensively restored the renal histology and functions during cisplatin-induced AKI. This may have been mediated by the inhibition of p38 MAPK signaling and TNFα production, blocking the inflammatory response and reducing cell apoptosis. These findings demonstrate that DEPTOR is a critical mediator in AKI. Future work must examine whether *DEPTOR* deficiency plays a protective role in other organs, such as the liver or the ovary, during cisplatin treatment. If so, DEPTOR or its downstream targets (Supplemental Table 3 and 4, Supplemental Fig. 4) may be novel therapeutic targets. Importantly, as DEPTOR is strongly expressed in some cancers, including multiple myeloma, clinical suppression of DEPTOR expression or activity, or its downstream signaling pathway, may achieve the best combination for prevention of cisplatin-induced injury and cisplatin antitumor efficacy.

## Materials and methods

### Mice and mouse models

*PEPCK*–Cre mice (C57/BL6) were kindly provided by Professor Volker Haase (Vanderbilt University)^32^. For the *DEPTOR*-flox mice, ES Cell Clone (EPD0556_1_H09) was purchased from the European Conditional Mouse Mutagenesis Program (EUCOMM ID: 41725). Germline-transmitting chimeric mice were generated by Shanghai Model Organisms Center Inc. (Shanghai, China). *DEPTOR*^flox/+^ heterozygous mice were obtained by crossing into the C57BL/6 line. *DEPTOR*^flox/flox^ mice were crossed with the *PEPCK*–Cre mice to produce conditional *DEPTOR* knockout mice (*DEPTOR*^flox/flox^X^cre^Y, designated “cKO mice” in this study) and littermate control mice (*DEPTOR*^flox/flox^XY; Supplemental Fig. 1A). Tail DNA samples were used for genotyping with PCR using primers F1/R1. Gene deletion was detected in the kidney with PCR using primers F2/R2. The primer sequences are listed in Supplemental Table 1. All animals were randomly selected for experiments. All animal experiments were approved by the Southern Medical University Committee on the Use and Care of Animals and were performed in accordance with the Committee’s guidelines and regulations.

To induce the AKI model in vivo, wild-type C57BL/6 mice or indicated transgenic mice aged 8–12 weeks were injected intraperitoneally with a single dose of 20 mg/kg cisplatin (Sigma), and those with 0.9% saline solution were used as the controls. The wild-type mice were killed on days 1–3 after cisplatin administration and the transgenic mice on day 3.

### Evaluation of kidney functions

BUN, serum creatinine, and the urinary protein/creatinine ratio were measured with the IDEXX Catalyst Chemistry Analyzer (IDEXX Laboratories).

### Histology, immunohistochemistry, and immunofluorescence

Kidney samples were fixed in 10% neutraformaline and embedded in paraffin, and 4 μm sections were stained with PAS. Histological injury in the cortex was evaluated independently by two histologists and was scored as previously described, by counting the percentage of tubules that displayed tubular necrosis, cast formation, or tubular dilation, as follows: 0, normal; 1, <10%; 2, 10–25%; 3, 26–50%; 4, 51–75%; 5, >75% (Ref. 37). Ten fields (×200 magnification) were randomly selected and counted per kidney.

IHC staining was performed with a standard procedure, using a horseradish-peroxidase-conjugated anti-immunoglobulin G secondary antibody (Jackson ImmunoResearch) visualized with 3, 3′-diaminobenzidine. The slides were counterstained with hematoxylin.

Immunofluorescent staining was performed as for IHC, except that the secondary antibody was labeled with Alexa Fluor 594 or 488 (Molecular Probes), and 4′,6-diamidino-2-phenylindole (DAPI; Molecular Probes) was used to visualize the nuclei. Proximal tubules were stained with fluorescein isothiocyanate-labeled LTA (Vector Laboratories). Immunofluorescence images were obtained with FluoView FV1000 confocal microscopy (Olympus). The antibodies used for the different experiments are described in Supplemental Table 2.

### Western blotting analysis

The renal cortex tissues were triturated, lysed on ice, and then boiled in SDS loading buffer. The protein extracts were subjected to 6–12% SDS-PAGE and then processed with a standard protocol.

### Cell apoptosis assay

Cell apoptosis was evaluated in kidney sections with a TUNEL assay using the DeadEnd Fluorometric TUNEL System (Promega).

### qPCR assay

Total RNA was extracted from the renal cortex tissues and reverse-transcribed to cDNA. A qPCR analysis was performed with the RealStar SYBR Green kit (Genstar BioSolutions) on the StepOnePlus™ Real-Time PCR System (Applied Biosystems). The relative expression of each gene was normalized to that of *Gapdh* with the 2^−ΔΔCt^ method. The quantitative real-time PCR (qPCR) primer sequences are listed in Supplemental Table 1.

### TNFα quantification with ELISA

The level of TNFα protein in the renal cortex was quantified with the mouse TNFα enzyme-linked immunosorbent assay (ELISA) kit (Nanjing Jiancheng Bioengineering Institute).

### Cell culture and treatment

HK-2 cells were cultured in RPMI-1640 medium and HEK293 cells in Dulbecco’s modified Eagle’s medium, with 10% fetal bovine serum. The HK-2 and HEK293 cells were transfected with *DEPTOR*-specific or control siRNA (GenePharma) using Lipofectamine RNAiMAX Reagent (Invitrogen). The pRK5 FLAG DEPTOR plasmid was a gift from David Sabatini (Addgene plasmid #21334; Ref. 13), and the pcDNA3.1-p38α MAPK plasmid was purchased from Genechem, and were used to transfect HK-2 and/or HEK293 cells with Lipofectamine 3000 Reagent (Invitrogen). To induce AKI in vitro, the cells were incubated with 20 μM cisplatin for 24 h.

### Statistical analysis

Data are expressed as means ± SEM. Quantification data for western blotting and qPCR are presented as % of control to make a straightforward comparison and control for unwanted sources of variation. Differences between groups were analyzed with an independent *t-*test (SPSS software, version 13.0; SPSS Inc., Chicago, IL) or, if the data violated a normal distribution, by a nonparametric Mann–Whitney test. *P* < 0.05 is considered statistically significant.

## Electronic supplementary material

Supplemental materials
