# Supplementary material for: Loss of DEPTOR in renal tubules protects against cisplatin-induced acute kidney injury
Source: Cell Death Dis. 2018 Apr 18;9(5):441. doi: 10.1038/s41419-018-0483-3 (PMC5906659; doi:10.1038/s41419-018-0483-3)

Supplemental Materials

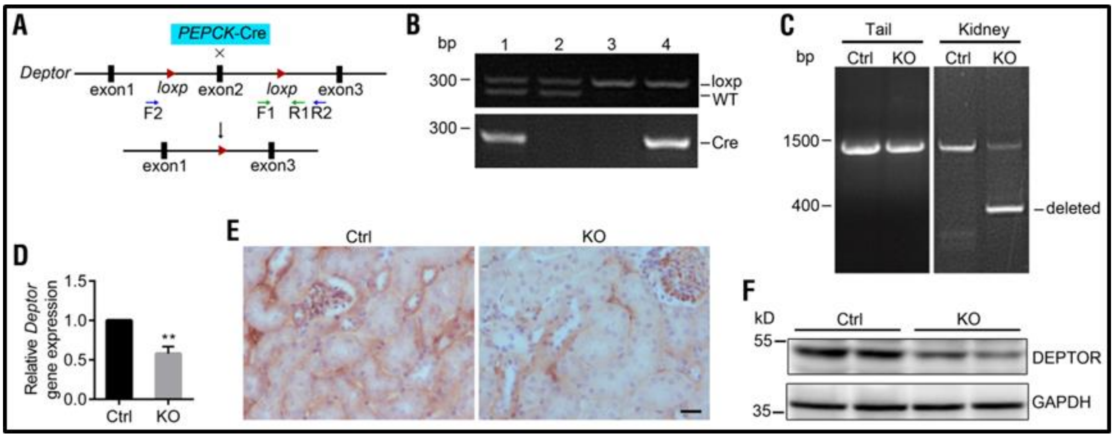

Supplemental Figure 1. Generation of mice with *DEPTOR* specifically deleted in renal proximal tubules. (A) Strategy for the production of *DEPTOR* cKO mice. *DEPTOR* flox mice were crossed with *PEPCK*-Cre mice to produce *DEPTOR* KO mice, in which the second exon of *DEPTOR* gene was deleted. F1/F2: forward primers for PCR identification, R1/R2: reverse primers for PCR identification. (B) Genotyping the F2 offspring by PCR with primers F1/R1 after mating the transgenic mice. M: DNA marker. (C) Identification of gene deletion in renal genomic DNA by PCR with primers F2/R2. Tail genomic DNA was used as the endogenous control. (D) qPCR analysis showed DEPTOR mRNA levels in the renal cortex of KO mice were obviously reduced compared with control mice. Data are expressed as mean values ± SEM (n = 5). ***P* <0.01. (E) IHC staining showed DEPTOR expression was significantly reduced in renal proximal tubules of *DEPTOR* KO mice compared with control mice. Scale bar = 20 μm. (F) Western blotting analysis showed DEPTOR expression in the renal cortex of KO mice was decreased.

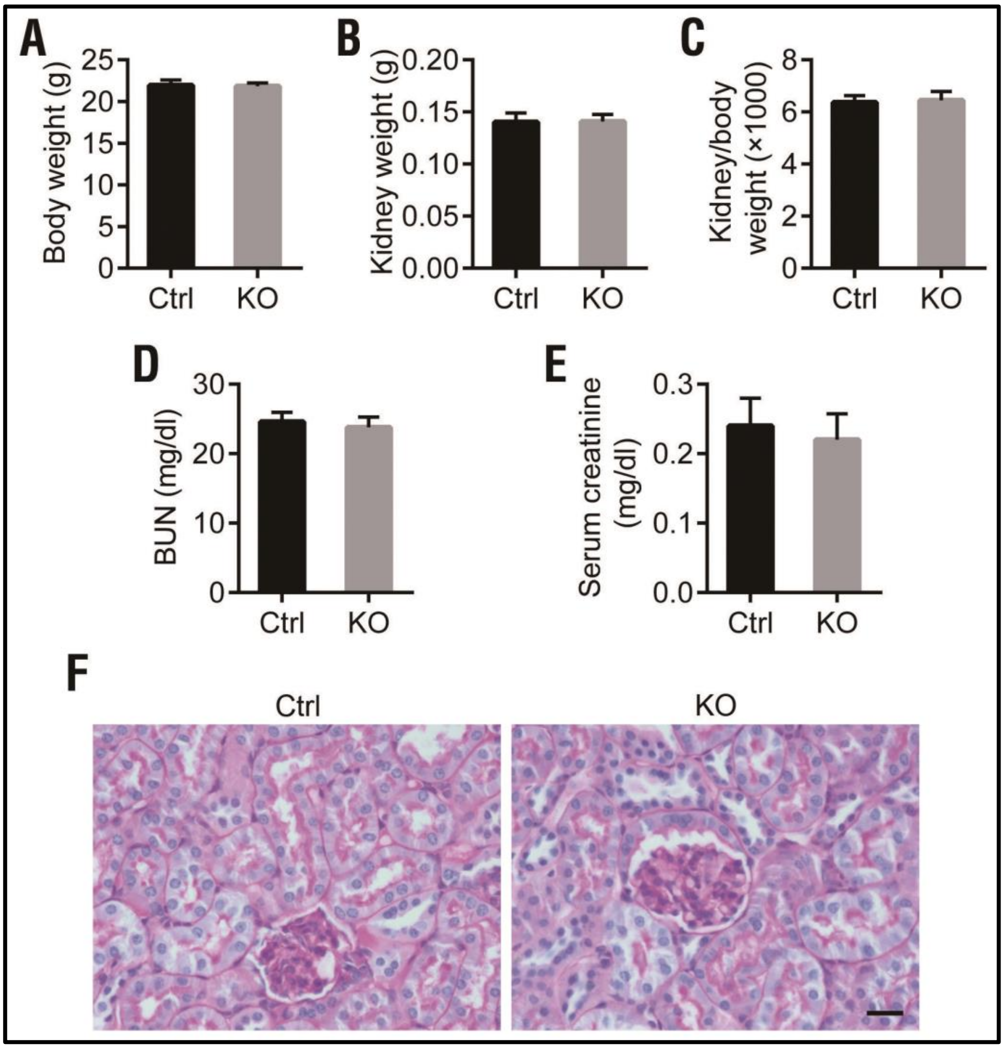

Supplemental Figure 2. Normal renal functions and histology in *DEPTOR* KO mice under physiological conditions. No significant difference was observed in bodyweight (A), kidney weight (B), kidney/body weight ratio (C), BUN (D), or serum creatinine (E) between *DEPTOR* KO and control (Ctrl) mice. BUN and creatinine were measured with the IDEXX Catalyst Chemistry Analyzer. (F) Kidney histology of paired mice detected with PAS staining. Mice were 8-12 weeks old. Scale bar = 20 μm. Bars indicate mean values ± SEM. *n* = 8.

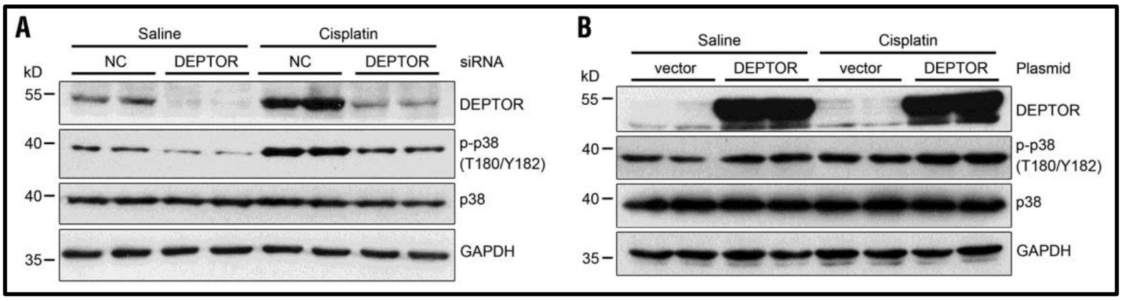

Supplemental Figure 3. DEPTOR regulates p38 MAPK activity in HEK293 cells. (A) Immunoblotting showed that *DEPTOR* knockdown by siRNA inhibited p38 MAPK activity in HEK293 cells, with or without cisplatin treatment. In the cisplatin group, cells were incubated with 20 μM cisplatin for 24 h, the same below. (B) Immunoblotting showed that *DEPTOR* overexpression enhanced p38 MAPK activity in HEK293 cells.

Supplemental Table 1. Primers used for experiments

Gene	Forward primers(5'–3')	Reverse primers(5'–3')
DEPTOR - *F1/R1*	GGAAGTGAAGACCTGTGAAGA TAAGAG	CAGTCTAGAAAAGCAGATAACC CAAGTA
DEPTOR - *F2/R2*	CGTCGAGAAGTTCCTATTCCGA AG	GTGTGCCATTGAAATTTGTTACC AT
PEPCK–Cre	GCATAACCAGTGAAACAGCATT GCTG	GGACATGTTCAGGGATCGCCAG GCG
DEPTOR– qPCR	AGCAGAGAGAGCTGGAACGC	CAGAGGCCTCCTTATGTTCA
Mouse Kim1	ATGCCCATCTTCTGCTTGTCA	CCTTGTAGTTGTGGGTCTTCT
Mouse NGAL	TGGCCCTGAGTGTCATGTG	CTCTTGTAGCTCATAGATGGTGC
Mouse TNFα	CCTGTAGCCCACGTCGTAG	GGGAGTAGACAAGGTACAACCC
Mouse Gapdh	TGTGTCCGTCGTGGATCTGA	TTGCTGTTGAAGTCGCAGGA
Human TNFα	CCTCTCTCTAATCAGCCCTCTG	GAGGACCTGGGAGTAGATGAG
Human Gapdh	ACAACTTTGGTATCGTGGAAGG	GCCATCACGCCACAGTTTC

Supplemental Table 2. Antibodies used for experiments

Antibody	Vendor	Catalog number	Application	Working dilution for WB or (IF/IHC)
Rabbit anti-DEPTOR	Cell Signaling Technology	11816	WB	1:2000
Mouse anti-DEPTOR	Santa Cruz Biotechnology	sc-398169	WB,IHC	1:2000 (1:100)
Rabbit anti-p-S6 (S235/236)	Cell Signaling Technology	4858	WB, IHC,IF	1:2000 (1:200)
Mouse anti-S6	Santa Cruz Biotechnology	sc-74459	WB	1:2000
Rabbit anti-p-Akt (S473)	Santa Cruz Biotechnology	sc-7985-R	WB	1:2000
Mouse anti-Akt	Cell Signaling Technology	2920	WB	1:2000
Rabbit anti-p-p38(T180/Y182)	Cell Signaling Technology	4511	WB	1:2000
Rabbit anti-p38	Cell Signaling Technology	8690	WB	1:2000
Rabbit anti-Ki67	Cell Signaling Technology	9129	IF	1:200
Rabbit anti-PARP	Cell Signaling Technology	9532	WB	1:500
Ribbit anti- GAPDH	Sungene	KM9002T	WB	1:5000
Rabbit anti-p-ERK1/2(T202/Y204)	Cell Signaling Technology	4370	WB	1:2000
Rabbit anti-ERK1/2	Cell Signaling Technology	4695	WB	1:2000
Rabbit anti-p-JNK(T183/Y185)	Cell Signaling Technology	4668	WB	1:2000
Rabbit anti-JNK	Cell Signaling Technology	8690	WB	1:2000
Rabbit anti-p53	Abclonal	A3185	WB	1:2000
Mouse anti-F4/80	Santa Cruz Biotechnology	sc-377009	IF	1:50

Supplemental Table 3. List of 46 genes with significant alteration in the mRNA level in the renal cortex of the *DEPTOR*-cKO mice *vs* that of the control mice under physiological condition by transcriptomic sequencing and screening assay.

Gene	log_2 (Fold change)	Description
Cyp2b10	-4.01	cytochrome P450, family 2, subfamily b, polypeptide 10
Ccl28	-3.68	chemokine (C-C motif) ligand 28
Rnf180	-3.41	ring finger protein 180
Rn7sk	-3.25	RNA, 7SK, nuclear
Rmrp	-3.02	RNA component of mitochondrial RNAase P
Hsd11b1	-2.81	hydroxysteroid 11-beta dehydrogenase 1
Ctxn3	-2.80	cortexin 3
Slco1a1	-2.73	solute carrier organic anion transporter family, member 1a1
Srd5a2	-2.61	steroid 5 alpha-reductase 2
Acsm3	-2.59	acyl-CoA synthetase medium-chain family member 3
Cyp4a12a	-2.56	cytochrome P450, family 4, subfamily a, polypeptide 12a
Rpph1	-2.48	ribonuclease P RNA component H1
Hapln1	-2.36	hyaluronan and proteoglycan link protein 1
Kif20b	-2.34	kinesin family member 20B
Eri2	-2.32	exoribonuclease 2
Pik3c2g	-2.29	phosphatidylinositol 3-kinase, C2 domain containing, gamma polypeptide
Mir6236	-2.25	microRNA 6236
Hprt	-2.17	hypoxanthine guanine phosphoribosyl transferase
Zpld1	-2.16	zona pellucida like domain containing 1
Mfsd2a	-1.97	major facilitator superfamily domain containing 2A
Odc1	-1.96	ornithine decarboxylase, structural 1
Frzb	-1.93	frizzled-related protein
Ces2b	-1.87	carboxyesterase 2B
Slc22a7	-1.80	solute carrier family 22 (organic anion transporter), member 7
Lipo1	-1.72	lipase, member O1
Nr4a1	-1.70	nuclear receptor subfamily 4, group A, member 1
Azgp1	-1.66	alpha-2-glycoprotein 1, zinc
Il34	-1.66	interleukin 34
Egr1	-1.63	early growth response 1
Cxcl12	-1.57	chemokine (C-X-C motif) ligand 12
Ttbk1	-1.55	tau tubulin kinase 1
Cd36	-1.54	CD36 antigen
Ces2c	-1.35	carboxylesterase 2C
Tnfsf10	-1.26	tumor necrosis factor (ligand) superfamily, member 10

B4galt5	-1.26	UDP-Gal:betaGlcNAc beta 1,4-galactosyltransferase, polypeptide 5
C1qtnf3	-1.20	C1q and tumor necrosis factor related protein 3
Scd1	1.35	stearoyl-Coenzyme A desaturase 1
Apoe	1.39	apolipoprotein E
Pdk4	1.49	pyruvate dehydrogenase kinase, isoenzyme 4
Cish	1.49	cytokine inducible SH2-containing protein
Tma7	1.88	translational machinery associated 7
B4galnt1	1.94	beta-1,4-N-acetyl-galactosaminyl transferase 1
Kynu	2.30	kynureninase (L-kynurenine hydrolase)
Cyp4a14	2.76	cytochrome P450, family 4, subfamily a, polypeptide 14
Prlr	3.14	prolactin receptor
Gpr112	3.70	adhesion G protein-coupled receptor G4

Supplemental Figure 4. Network for protein interactions among the 46 genes.

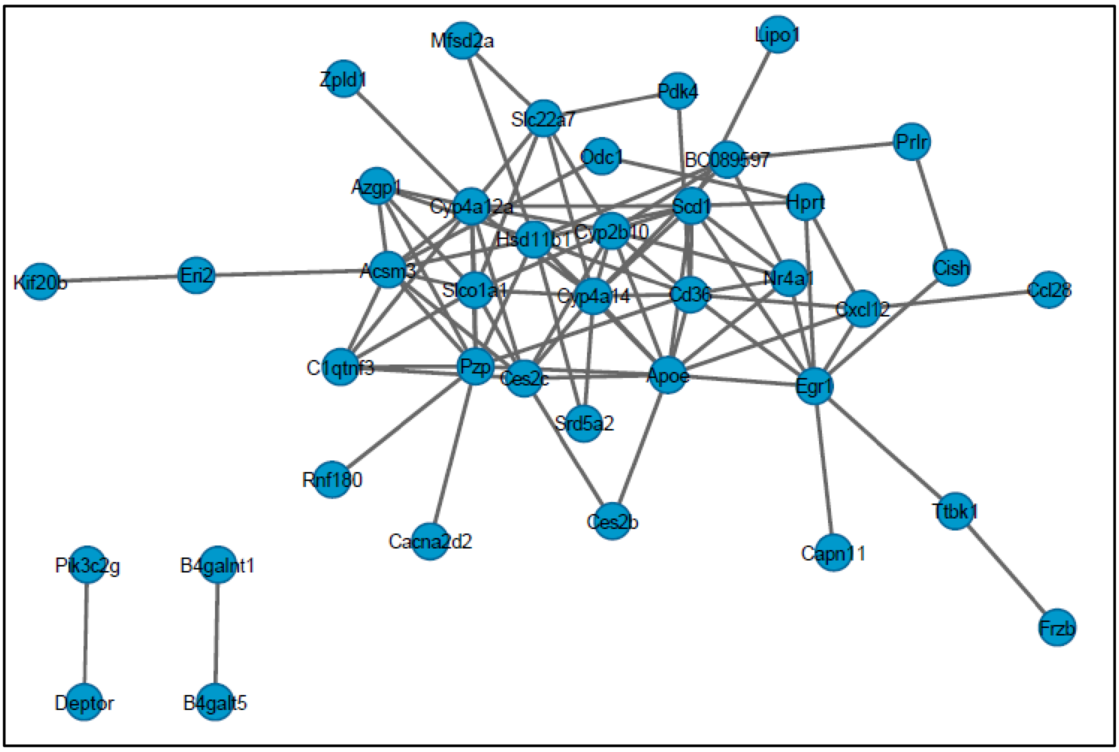

Supplement: Supplementary file 1 — Supplemental materials [file 41419_2018_483_MOESM1_ESM.pdf]
